# ‘Message to Dentist’: Facilitating Communication with Dentally Anxious Children

**DOI:** 10.3390/dj7030069

**Published:** 2019-07-01

**Authors:** Helen Rodd, Laura Timms, Fiona Noble, Sarah Bux, Jenny Porritt, Zoe Marshman

**Affiliations:** 1 School of Clinical Dentistry, University of Sheffield, Sheffield S10 2TA, UK; 2 Department of Paediatric Dentistry, Charles Clifford Dental Hospital, Sheffield S10 2SZ, UK; 3 Clapton Dental Practice, London E5 0LH, UK; 4 Department of Psychology, Sociology and Politics, Sheffield Hallam University, Sheffield S1 1WB, UK

**Keywords:** dental anxiety, communication tool, paediatric dentistry, cognitive behavioural therapy

## Abstract

Dental anxiety affects children worldwide and can have negative consequences on oral health. This study aimed to evaluate a novel communication aid ‘message to dentist’ (MTD), as part of a wider cognitive behavioural therapy approach to reduce dental anxiety in young patients. Dentally anxious children, aged 9–16 years, were invited to complete the MTD proforma, before and following their course of treatment. They scored how worried they were and their anticipated pain levels on a scale of 1–10 (10 being the worst outcome). They also wrote down their coping plans and post-treatment reflections. One hundred and five children, from a UK general dental practice and a hospital clinic, were included. They had a mean age of 11.6 years, and 65% were female. There was a significant reduction in self-report worry (from 4.9 to 2.1) and anticipated pain (from 5.1 to 2.0) scores (*p* < 0.05, paired *t*-test). Many children (30%) used listening to music/audiobook as a coping strategy. Thematic analysis revealed concerns around pain, uncertainty, errors and specific procedures. The MTD proforma proved an effective means of facilitating communication between anxious children and the dental team, allowing them to identify their worries and make personalised coping plans.

## 1. Introduction

The provision of dental care for children who have dental anxiety is a universal and, seemingly, perpetual problem. Recent worldwide data suggest that up to 30% of children report moderate levels of dental anxiety, which may have negative impacts for the individual, their family, dental health professionals and society as a whole [1,2,3]. Notably, children who are dentally anxious tend to have poorer oral health (higher caries experience and extractions) and are symptomatic or irregular users of dental services [1,2,3]. It therefore remains imperative that anxiety management is integral to routine dental care for all at-risk children, in the hope of reducing a lifelong fear of dentistry and poor oral health outcomes.

There is an extensive literature on the numerous behavioural techniques that can be employed to ‘manage’ children’s dental anxiety [4,5,6]. However, in recent years there has been growing interest in the role of psychological approaches, such cognitive behavioural therapy (CBT), for not just ‘managing’ children’s dental anxiety, but reducing it in both the short and longer-term [7]. CBT has a good evidence base for reducing a variety of paediatric anxiety disorders and associated co-morbidities [8,9]. It is described as talking therapy, helping individuals to understand their ‘problem’ better, and develop positive coping strategies to change unhelpful thoughts, feelings and behaviours. A 2018 systematic review, based on six randomised controlled trials, reported on the effectiveness of CBT in reducing child dental anxiety [7]. Several of these studies involved psychologist-led CBT, which is a high-intensity and costly approach. However, Porritt and colleagues have described the development and evaluation of a lower intensity guided self-help intervention based on the Five Areas Model of CBT [10,11]. This intervention, developed with the input of children themselves, is called ‘Your teeth you are in control’, and is aimed at 9–16 year-old children with mild to moderate levels of dental anxiety. Following an initial assessment, children deemed as anxious are provided with a written or online guide that incorporates an interactive communication aid called a ‘message to dentist’ (MTD) proforma. The purpose of the MTD proforma is to encourage the child to inform the dentist of their specific worries and planned coping strategies. The proforma is also utilised after every treatment episode to stimulate self-reflection and build positive memories.

This research team previously reported a significant reduction in child anxiety (using the Modified Child Dental Anxiety Scale) following the use of the ‘Your teeth you are in control’ intervention [10]. The paper aims to describe findings specifically relating to the MTD communication aid proforma in more detail than has been previously reported. The objectives were to: (i) Compare self-report pre- and post-treatment pain and worry scores; (ii) determine any differences in these scores for children seen in primary or secondary care settings and according to gender; (iii) gain insight into children’s specific dental fears, coping strategies, self-reflection and rewards.

## 2. Materials and Methods

### 2.1. Participants

Children who completed the MTD proforma as part of the initial development and evaluative study in the dental hospital setting (n = 40) were considered research participants and were subject to full ethical approval and research governance procedures (REC 13/YH/0163) [10]. However, following this initial evaluation of the CBT intervention, ‘Your teeth you are in control’, and the MTD proforma have all been used as part of routine care for anxious patients within the paediatric dentistry clinic of the Charles Clifford Dental Hospital, Sheffield, UK and within a National Health Service general dental practice in London. Anonymised data from these patients have been collected as part of an ongoing service evaluation of the newly introduced CBT pathways. Parents provided consent for these patients to receive the CBT pathway and complete the MTD proforma, but they were not considered as research participants. Data collection for patients seen in general practice commenced in 2017 and is ongoing, whereas data collection for children seen in the hospital setting commenced in 2015 and is also still ongoing.

**Figure 1:**
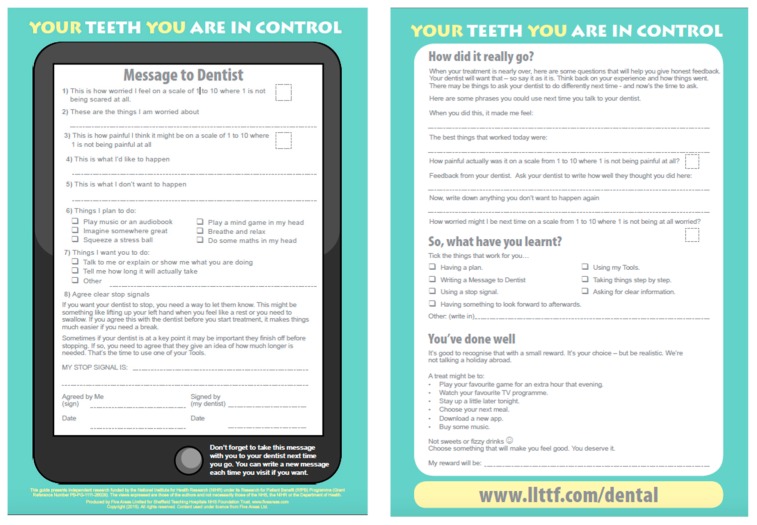
The ‘message to dentist’ proforma, which is given to the child in an A4 2-sided format (a pdf of ‘message to dentist’ is free to download at www.llttf.com/dental).

Children who were seen in the general dental practice or the hospital clinic, who were aged between 9–16 years, and who appeared to be dentally anxious (e.g., crying, refusing treatment, reporting fear of injections or other procedure) were offered the ‘Your teeth you are in control’ guide, which incorporated the MTD proforma. An example of a completed MTD is shown in Figure 1 below.

From Figure 1, it can be seen that the MTD proforma prompts the child to answer the following key questions to facilitate further discussion with their clinical care team.

Pre-treatment, children document:

- Self-report worry score from 1–10 (10 = most worried);
- Things which they are worried about;
- Self-report anticipated pain score from 1–10 (10 = worst pain);
- Items of care that they are willing to accept;
- Things that they do not want to happen;
- Things they plan to do, to help them cope (from a list of suggested options);
- An agreed stop signal (signed by patient and clinician);
- Things that worked well;
- Self-report worry score from 0–10 (10 = most worried);
- Self-report anticipated pain score from 0–10 (10 = worst pain);
- ‘Reward’ for progress made.

Other factors supporting the use of the MTD were: The child did not require urgent treatment; could be scheduled to the same clinician for the course of treatment, and had adequate levels of literacy and cognition to complete the MTD (with support if necessary). Children were told the rationale for the use of the MTD—with the intention to help them feel less anxious about visiting the dentist and/or specific dental procedures. Parents were also given written ‘tips’ for supporting their child at subsequent dental visits. Children saw one clinician in the general dental practice (SB) who had no post-graduate training in paediatric dentistry, whereas children in the hospital setting saw a variety of specialist paediatric dentists, all of whom were trained in the application of the intervention.

Children were asked to complete the MTD proforma prior to their subsequent visit, and it was mutually agreed what would happen at this next visit (e.g., a simple preventive procedure, radiographs). At this second appointment, their responses on the MTD proforma were discussed and acknowledged by their dentist (or therapist), and a plan agreed. If the child had forgotten their MTD proforma, or had not completed it, they were invited to fill in the proforma together with their clinician. On completion of their course of treatment (which could include sedation or general anaesthesia if necessary), children were asked to use the MTD proforma to reflect on their current anxiety and pain scores, appraise how treatment had gone, as well as describing the rewards they had planned for themselves. This activity was undertaken in the dental surgery with appropriate input and encouragement from the dental team and parents/carers.

### 2.2. Data Processing and Analysis

Numerical and qualitative data were extracted from the MTD proforma as described below.

Numerical data (pain and anxiety scores) were entered into the Statistical Package for Social Sciences (SPSS v23, IBM statistics). Data were found to be normally distributed and *t*-tests (independent or paired as appropriate) were used to determine any statistically significant differences in self-report pain and anxiety scores between: (i) Initial visit and on completion of a course of treatment; (ii) practice and hospital patients, (ii) girls and boys. The level of significance was set at *p* < 0.05.

Children’s proposed coping strategies were coded, entered in SPSS, and subject to simple descriptive analysis.

Qualitative data were initially entered into an Excel dataset (Microsoft Office v7) and were subject to simple thematic analysis by four investigators, independently [12]. Four main areas were analysed as dictated by the MTD proforma: Things children were worried about; what they would like to happen; what went well, and their planned reward. Within these main areas, the investigators familiarised themselves with the data to identify themes and extracted data, into table form, to support their findings. The four investigators then compared the themes they had identified, and any disagreements in interpretation were resolved through discussion.

## 3. Results

### 3.1. Participants

Message to dentist data were obtained for a total of 105 children; 53 had been seen in the hospital clinic, and 52 had been seen in general dental practice. Patients had a mean age of 11.6 years (SD = 1.98; range = 8–16 years), and there was a higher percentage of girls than boys (65% versus 35%). There were no significant differences in the mean age of children, or the proportion of female/male patients, between the two clinical settings. None of the children in the dental practice underwent inhalation sedation (IS) or general anaesthesia (GA) as part of their treatment. However, within the hospital cohort, 41 (71%) received IS on at least one visit and 3 (6%) required a GA.

### 3.2. Worry and Pain Scores

#### 3.2.1. Pre- and post-treatment

As shown in Table 1, the mean pre-treatment worry score was 4.9 (SD = 2.34; range = 1–10). On completion of their course of treatment, children reported being significantly less worried, with a mean score of 2.1 (SD = 1.69; range = 0–8; *p* < 0.001, paired *t*-test). This mean drop of 2.8 points was considered sizable although, within the cohort, there were still some children who were very anxious, rating their worry levels as 8 out of a possible 10. The mean anticipated pain score, before any treatment, was 5.1 (SD = 2.40; range = 1–10). This also showed a significantly significant decrease on completion of the course of treatment to a mean of 2.0 (SD = 1.93; range = 0–6). It should be noted, that although the MTD proforma asked the children to score from 1 to 10 (10), some children did score themselves as zero, and this was analysed as such.

#### 3.2.2. Hospital and practice patients

Children seen in the hospital setting reported significantly higher initial worry scores (mean = 6.0; SD = 1.95; range = 2–10) than those seen in general dental practice (mean = 3.7; SD = 2.15; range = 1–9) (*p* < 0.001, independent *t*-test). Similarly, initial self-report anticipated pain scores were significantly higher in the hospital patient group (mean = 6.2; SD = 1.87; range = 2–10) than in the practice group (mean = 4.0; SD = 2.37; range = 1–10) (*p* < 0.001, independent *t*-test). Following treatment, mean worry scores remained significantly higher in the hospital group than the practice group (4.0 versus 1.8, *p* < 0.05), but there was no significant difference for anticipated pain scores according to the setting. Although hospital patients had higher scores overall, both hospital and practice patients showed significant reductions in self-report worry and pain scores following treatment (*p* < 0.05, paired *t*-test).

#### 3.2.3. Gender

There were no statistically significant differences in mean pre-treatment pain or worry score according to gender (raw data not shown).

### 3.3. What Children Said They Were Worried About

Thematic analysis of the children’s free text, documented on their MTD proformas, revealed four themes relating to things they were reportedly worried about at their initial visit. These are illustrated in Table 2 and included a fear of: Experiencing pain; something bad happening (catastrophising); not knowing what was happening (uncertainty); and specific procedures. Examples of children’s responses are provided to illustrate these four themes. Needles and injections were commonly reported as specific ‘procedures’ that children were anxious about, and there was some overlap with the theme of pain ‘*I think the injection is going to hurt.*’ Within the overall theme of catastrophising, children revealed a variety of concerns, relating to the dentist making a mistake. In some cases, children expressed worries that the wrong treatment would be performed, or more seriously, that the dentist would drop something, and the child would choke and die.

### 3.4. What Children Said They Would Like to Happen

Children used their MTD proforma to express their views on what they would like to happen at their future dental visit. Four themes were identified: An absence of pain; good communication; speed, and a positive clinical outcome. The positive outcome was seen to relate to both the management of the dental condition itself (having a ‘bad’ tooth filled or removed), as well as having a positive experience (everything going right and them leaving happy). Patients also hoped that the dental team would listen to them and tell them what was happening. Within the theme of good communication, children wanted to feel in control, by asking the dentist to cease treatment when necessary ‘*I would like to be able to say stop*’. They also expressed a strong preference for things to be done quickly and without incident. Children’s quotes are provided in Table 3 to illustrate these topics.

#### 3.4.1. Coping plans

Children could choose one or more coping plans from a suggested list on the MTD proforma. The most popular coping strategy (distraction), chosen by 30% of the sample, was to listen to music or an audiobook. Others opted to: Squeeze a stress ball/play with a fidget spinner (26%); do breathing and relaxation exercises (22%); play a mind game (9%); imagine being somewhere nice (8%), or do a maths puzzle in their head (7%).

#### 3.4.2. Stop signals

It was evident from the completed MTD proformas that all children (100%) and their dental team had engaged with the agreed stop signal and had signed the proforma to show their commitment to this. Invariably, this was a proposed hand signal: ‘*Lift up my hand’*; ‘*left hand in the air’*, and ‘*wave left hand*’. In some instances, children selected more unusual options, such as ‘*click my fingers*’ or ‘*shake my feet’*.

#### 3.4.3. Self-reflection

As part of their overall reflection, children were encouraged to write down, on their MTD proforma, the best things that had worked for them on that visit. Analysis of these data revealed three themes: Clinician competence; good communication and effective use of their own coping strategies (Table 4). Within the theme of clinician competence, children reflected on how quick the procedures had been and the actual absence of pain. Some children even stated that the injection had been the best thing that worked for them. Good communication between the child and the dentist was frequently referred to as being the best thing about the dental encounter. Children appeared to vary in how much information they did or did not want to hear, but that whatever their preference, the dentist had been reportedly mindful of their wishes. Having a plan and sticking to that plan was paramount to anxiety reduction and a positive experience. Children reflected that some of their planned coping strategies (as described earlier) had worked well for them, particularly listening to music.

#### 3.4.4. Rewards

On completion of their dental visit/treatment, children were invited to record on their MTD proforma what reward they had agreed with their parent/carer to recognise their achievements. The reward acts to positively reinforce the child’s engagement and progress made during the appointment. Children liked this opportunity, and it provided a nice prompt for discussion at the next appointment. These rewards were mostly found to relate to three themes: Use of digital technologies; social activities; and food-related treats. Examples of these are shown in Table 5 below.

## 4. Discussion

The use of the MTD communication aid proforma, as described in this paper, raises a number of clinically relevant points for further consideration. Firstly, the MTD proforma provided a straightforward means of recording a child’s overall pre-treatment anxiety and anticipated pain levels. Although several validated child self-report dental anxiety measures have been developed over the years, their use seems to be limited to research-related practice [1,13]. There is scant evidence to suggest that they are used routinely in primary, or even in specialist, dental care settings as part of a holistic assessment. Time constraints and misconceptions that drawing attention to a patient’s level of worry will only serve to increase anxiety are potential barriers to the routine assessment of anxiety. However, Dailey and colleagues reported the opposite finding in their study of adult patients seen in general dental practice [14]. Interestingly, a team in New Zealand has also developed a communication aid for children to improve their outcomes during a dental visit [15]. This self-report 26-item instrument, known as the Survey of Anxiety and Information for Dentists (SAID) was designed with children to capture, not just their anxiety levels, but also their oral health-related knowledge and coping preferences. Children were found to engage well with this tool, and it was important to them that they handed their completed questionnaire to the dentist personally. A subsequent electronic version, eSAID, was used in a randomised controlled trial with children aged 8–13 years in the UK [16]. The use of eSAID was found to significantly reduce pre-treatment anxiety, although it did not appear to improve children’s satisfaction, or cooperation. The use of the MTD proforma in the present study proved very helpful in revealing children’s individual and disparate fears about dentistry, providing an invaluable opportunity for the dental team to acknowledge and address them. It was interesting, for example, to see how catastrophising played a prominent role in maintaining some children’s anxiety. Children seemed particularly worried about something going wrong during their dental treatment, which is in keeping with findings from previous studies [17,18]. Armfield and Heaton also highlighted how ‘*fear of a medical catastrophe’* was a particular typology amongst dentally anxious adults [5]. Choking, suffocating and having ‘*bad reactions*’ to local anaesthetic were common concerns in this patient group. If the dental team remains unaware of these fear-provoking beliefs, patients will not receive appropriate and targeted interventions. For children, a fear of the dentist extracting the wrong tooth could be readily addressed by showing them a radiograph of the tooth to be removed, and demonstrating, with a mirror, which one it is in their mouth. Another example, revealed by the use of the MTD proforma, was a young girl whose dental anxiety related to a fear of germs. Once she was shown the cross-infection procedures that were adopted in the clinic, she was able to accept treatment. Without the MTD proforma, which revealed this specific fear to the clinician, normal behaviour management techniques may have failed to address the underlying cause of her anxiety.

Other simple, but effective, strategies for anxiety reduction used by patients were the use of individual coping tools and an agreed stop signal. The use of music was a popular coping choice, either using headphones or by playing music (of the child’s choice) aloud in the surgery. The biological and psychological benefit of music in reducing moderate anxiety in medical and dental settings is well recognised, although the evidence for the reduction of child dental anxiety has not yet been established [19]. The use of signalling has also been previously reported in the wider dental anxiety literature [5]. The use of a stop signal gives the patient a greater sense of control and predictability about the course of their treatment, which in turn helps with anxiety control. The MTD proforma incorporated a section to document the agreed stop signal, with signatures required from both the dental professional/s and child. Although it may seem obvious, the importance of trust and good communication in anxiety reduction cannot be overstated. The MTD proforma revealed many examples of how children valued being listened to, and being told what was going to happen. The importance of developing a strong therapeutic alliance between the child and dental the team has also been highlighted in previous studies [17,18]. Within the wider literature, there is a wealth of evidence to support the role of therapeutic relationships in paediatric anxiety reduction [20].

A novel aspect of the MTD proforma was the opportunity for children to reflect on their progress and to document how they had felt, what things had gone well, and what they had learnt? Children appeared surprised by how quickly and painlessly the procedures had been: ‘*It wasn’t painful,’ ‘It was faster than expected.’* The purpose of this feedback section was to stimulate self-reflection and build positive memories for children. The process of self-reflection and identifying how problems have been overcome may help increase children’s self-efficacy beliefs relating to their perceived ability to cope with treatment and any associated pain. Individuals with higher levels of pain self-efficacy beliefs have been found to experience less pain than those who report lower levels of pain self-efficacy beliefs, which highlights the importance of utilising CBT techniques that increase perceptions of mastery and confidence in paediatric patients [21]. There are several ways in which the MTD proforma may contribute to the more effective management of dental anxiety (e.g., enhancing trust, increasing sense of control, encouraging adoption of effective coping mechanisms, promoting parents’/professionals’ empathy and understanding). However, to date the MTD proforma has been used as part of the ‘Your teeth you are in control’ intervention, and therefore it is not possible to identify how this specific component of the intervention may contribute to the reduction of children’s dental anxiety. Future research would be required to evaluate whether the MTD proforma is an effective ‘stand-alone’ intervention, in the absence of the more comprehensive ‘Your teeth you are in control’ guide. Furthermore, data collection was conducted as part of a pragmatic service evaluation, rather than a research study and therefore details relating to patients who decided they did not want to complete the MTD proforma were not available, as logs were not kept of patients who do not wish to engage. However, clinical impressions would suggest that most children and families were very receptive to this as part of their routine care. The MTD proforma also proved extremely quick to complete, even within a general dental practice setting. The study did not identify any differences in self-report anxiety or anticipated pain scores according to gender, but differences according to age were not explored. An adequately powered study would be needed to consider the effect of other variables, such as age, or specific items of treatment provided, using more sophisticated statistical analysis. It is interesting to note that a high proportion of hospital participants were assessed as needing inhalation sedation (71%) as part of their care, which triangulates well with the children’s self-report anxiety scores. It would be interesting to conduct a longitudinal study of these children, to see if the MTD intervention reduced the need for IS in the future.

A final point to highlight was that children seen in general dental practice had significantly lower pre- and post-treatment self-report anxiety and pain scores than children seen in the hospital setting, although they still showed a significant reduction following the intervention. One of the most common reasons for children in the UK to be referred to specialist services is because of their reported anxiety and refusal of treatment with their own dentist. Referring dentists request that these children have sedation or even GA, for what is often simple treatment. Due to the escalating concerns about the numbers of children who are undergoing a GA for dental treatment, it is imperative that different approaches are sought for these vulnerable groups [22]. General dental care professionals have a fundamental role in identifying children with dental anxiety and proactively offering simple psychological interventions, before acute treatment needs arise [23]. Greater consideration and resources therefore need to be allocated to developing care pathways for children with dental anxiety, in the hope of reducing a lifelong fear of dentistry and reliance on pharmacological interventions.

## 5. Patents

The term Five Areas^TM^ is a registered trademark of Five Areas Resources Ltd. (www.fiveareas.com).

## Figures and Tables

**Table 1:** Descriptive statistics displaying self-reported pain and worry scores for children seen in primary and secondary care settings.

Patient Setting	Initial Worry Score	Post-Treat Worry Score	Initial Pain Score	Post-Treat Pain Score
Practice (n = 52)				
Mean (SD)	3.71 (2.15)	1.84 (1.55)	3.99 (2.37)	1.96 (1.97)
Range	1–10	0–6	1–10	0–8
Hospital (n = 53)				
Mean (SD)	5.98 (1.95)	4.00 (1.67)	6.21 (1.87)	1.75 (1.50)
Range	2–10	0–3	2–10	0–3
Total Group (105)				
Mean (SD)	4.85 (2.34)	2.06 (1.69)	5.12 (2.40)	1.95 (1.93)
Range	1–10	0–8	1–10	0–6

**Table 2:** Examples of things children reported being worried about, within four themes.

Pain	Catastrophising	Uncertainty	Procedures
For it to be painful	Something will go wrong	Not knowing what is happening	Metal stuff in mouth
For it to hurt	I am worried something bad will happen	I don’t really know what they are going to do	Water in mouth
Me screaming and getting hurt	The wrong tooth to be taken out	It might make me frustrated	Seat going back and having things in my mouth
I think the injection is going to hurt	Something might get stuck	It may take a long time	Drill
Pain and unable to eat	If the dentist makes a mistake	-	Just injections
-	-	-	Needles

**Table 3:** Examples of things children said they wanted to happen at their dental visit, within four themes.

Absence of Pain	Good Communication	Speed	Good Clinical Outcome
Not to hurt	The dentist to listen to me	It to go smoothly and quickly	For it to get sorted
So I could not feel the pain	Dentist to tell me what is going to happen	To be quick	For my teeth to be clean
Numbing gel	When I put my hand up they stop	To be as quick and painless as possible	Get tooth out so no more pain
I can’t feel much	To be treated kindly	All to go quick and fine	For them to take away the decay
-	-	Smooth and calm	Leave smiling

**Table 4:** Examples of things children said had worked best for them, following their dental visit, within three themes.

Clinician Competence	Good Communication	Use of Coping Strategies
The actual process, because it wasn’t painful	Showing me everything and what it did	Listening to music
It was faster than expected	Plan-knowing what will happen next	Hand signals
Nothing nasty went in my mouth	Not thinking about it and understanding what was going on	Mind games
Everything went well	The explanation so I knew what was happening	Music and stress ball
It was quick	Not too much information given	-
The injection	-	-
Topical numbing gel	-	-

**Table 5:** Rewards children selected for themselves, to recognise their progress, were categorised within three themes.

Digital Technology	Social Activities	Food-Related Treats
Play PS4	Friend coming to house	Popcorn
More screen time	Fun with cousins	Have ice cream
Playing on the computer	Play on trampoline	Chicken
Go on my phone	Football	Favourite food
To go on my iPod	-	Baking
Play station	-	-
